# Assessment of Comprehensibility of Industry Conflicts of Interest and Disclosures by Multiple Sclerosis Researchers at Medical Conferences

**DOI:** 10.1001/jamanetworkopen.2021.2167

**Published:** 2021-04-02

**Authors:** Sarah-Jane Martin, David P. J. Hunt

**Affiliations:** 1 Institute of Neurological Sciences, Queen Elizabeth University Hospital, Glasgow University, Glasgow, United Kingdom; 2 Anne Rowling Clinic, Centre for Clinical Brain Sciences, University of Edinburgh, Edinburgh, United Kingdom

## Abstract

This cross-sectional study uses length of disclosure slide display times and slide word counts to assess the comprehensibility of industry conflicts of interest (COI) disclosures by researchers presenting at the world’s largest multiple sclerosis (MS) conference.

## Introduction

Financial relationships between health care professionals and the pharmaceutical industry should be transparent, especially in influential arenas such as medical conferences. Typically, a disclosure slide is mandated at the start of every conference presentation to inform the audience of the presenter’s conflicts of interest (COIs). However, the comprehensibility of this disclosure is dependent on the transparency behavior of the presenter, who decides how long the slide—together with its COI information content—is displayed.

The potential for industry payments to influence physician behavior has elicited concern in many fields of health care.^1^ The field of multiple sclerosis research has recently come under particular scrutiny. Concerns have been expressed regarding industry influence on researcher behavior^2^ in the context of dramatic drug cost increases not seen in other specialties.^3^ We therefore measured the association between the intensity of industry COIs and the transparency behavior among individuals who delivered oral presentations at the world’s largest multiple sclerosis conference.

## Methods

We conducted a cross-sectional study to analyze the information content of the COI disclosure slide at platform presentations at the Congress of the European Committee for Treatment and Research in Multiple Sclerosis (ECTRIMS) in Stockholm, Sweden, in 2019. Using the information displayed in the slide presented in the ECTRIMS Online Library, we recorded the total number of companies in which the presenter declared a personal COI (eMethods in the Supplement). We determined the intensity of the COI by classifying individual presenters’ COIs into the following categories: “no conflicts” (no COIs), “some conflicts” (≥1 COI but <10), and “heavy conflicts” (≥10 COIs). We then assessed the following transparency behaviors for each presentation: (1) the length of time the disclosure slide was displayed, compiled by one author (S.-J.M.) using the video player time stamp and the second author (D.P.J.H.) using a stopwatch, and (2) the word count of the slide, compiled either by manual counting or use of the word count function in Word (Microsoft Corp) for disclosures longer than 100 words. This study involved analysis of publicly available datasets only. As such, research ethics committee review was not required, following the guidance and regulations of the NHS Health Research Authority. This study followed the Strengthening the Reporting of Observational Studies in Epidemiology (STROBE) reporting guideline.

Physicians based in the United States are mandated to declare details of industry payments. We used the Open Payments search tool from the Centers for Medicare and Medicaid Services to investigate the mean value of each declared industry conflict for US-based conference speakers for the 3 years before the 2019 conference. The correlation between the number of COIs and length of display time of the disclosure slide was calculated using the Spearman rank correlation coefficient. Comparison of the proportion of readable disclosures between COI groups was performed using a χ^2^ test for trend. Statistical analyses, descriptive statistics, and linear regression were performed using Prism version 8 (GraphPad). The level of statistical significance was set at 2-sided *P* < .05.

## Results

**Figure 1.**
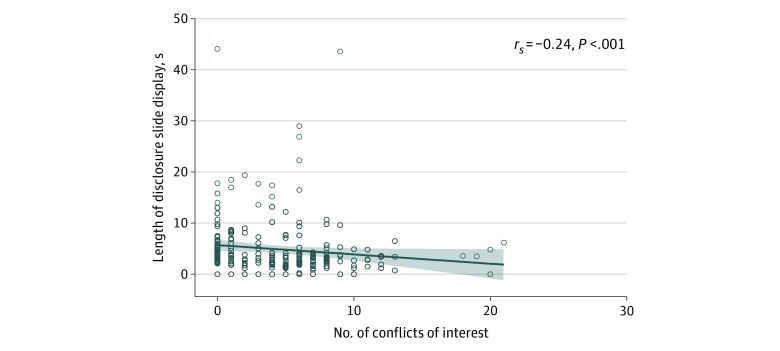
Intensity of Industry Conflicts of Interest and Length of Disclosure Slide Display Time at an International Multiple Sclerosis Conference An inverse correlation existed between the number of industry conflicts of interest and the length of display times of the disclosure slide. Each circle represents an individual presentation (n = 240). The solid blue line represents the estimated linear association, with 95% CI (shaded section).

We assessed 240 oral presentations at the 2019 annual ECTRIMS Congress. Of these presentations, 57 (24%) were delivered by researchers with no conflicts, 160 (66%) by researchers with some conflicts, and 23 (10%) by researchers with heavy conflicts. We found an inverse correlation between the number of COIs of the presenter and length of display time of the disclosure slide (*r_s_* = −0.24; *P* < .001) (Figure 1).

**Figure 2.**
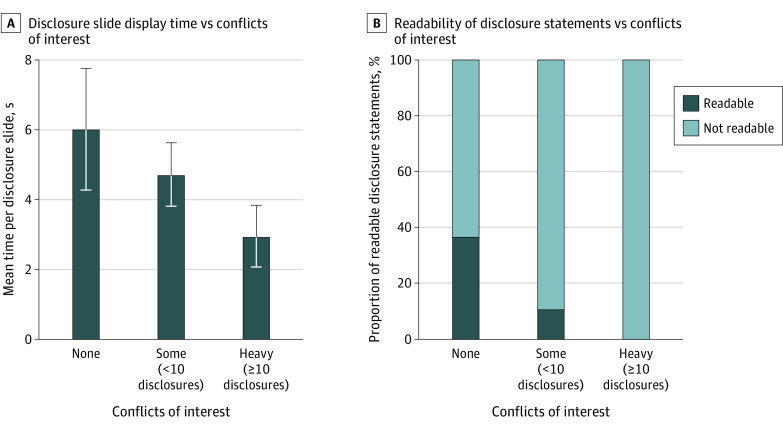
Display Time and Comprehensibility of Disclosure Statements A, Mean length of display time of disclosure slide by conflicts of interest category. Error bars indicate 95% CIs. B, Proportion of readable disclosure statements by conflicts of interest category.

We then assessed whether the length of display time of the disclosure slide was sufficient for meaningful comprehension of the slide contents. The mean time of display for the disclosure slide was 6.0 seconds in talks when the presenters had no conflicts (95% CI, 4.3-7.8 seconds; Figure 2A), 4.7 seconds for those with some conflicts (95% CI, 3.8-5.6 seconds), and 2.9 seconds for those with heavy conflicts (95% CI, 2.1-3.9 seconds). To assess whether these slide display times were sufficient for full comprehension of the presenter’s conflicts of interest, we classified a disclosure slide as “readable” if the total number of words in the slide could be read in full at the average reading speed (3.8 words/s).^4^ Disclosure statements exhibited at faster rates were classified as “not readable.” Of the 57 presentations by individuals with no conflicts, 21 (37%) were readable compared with 18 of 160 presentations (11%) by individuals with some conflicts, and none of the 23 presentations by individuals with heavy conflicts (χ^2^ for trend = 23; *P* < .001) (Figure 2B). The mean disclosure slide reading rate from the group with heavy (≥10) conflicts was approximately 40 words/s, more than 10 times faster than the average reading speed.^4^ For the 31 US-based physicians who were presenters at this conference, there was a strong correlation between the number of declared COIs and the amount of personal payments from industry (*r*_s_ = 0.77; *P* < .001). The mean financial value of each declared COI was $23 500.

## Discussion

Conflicts of interest with the pharmaceutical industry should be clearly declared. This study’s findings suggest an inverse association between transparency behavior at conferences and the degree of personal COIs. Presentations from individuals with strong links to industry might be anticipated to require a longer time to explain their disclosures. However, our analysis shows that such presentations are associated with shorter slide display times, resulting in a speed of delivery that almost always prevents adequate comprehension.

Industry conflicts have been shown to influence individual physician behaviors, such as prescription of brand name drugs.^1^ The findings of this cross-sectional study suggest that such conflicts may also have the potential to influence other behaviors, resulting in reduced transparency. We used the number of personal industry conflicts as a primary measure of the level of industry influence, rather than the financial value of industry payments. This limitation arose because many speakers outside the United States are not legally required to declare the value of these transactions. Further limitations of our study were a potential bias toward European speakers and restriction of the analysis to presenters at a single international conference.

Concerns about transparency and disclosure comprehensibility have been raised across medical specialties.^5,6^ Together, these findings raise serious questions about the adequacy of the current disclosure system at medical conferences. Simple, standardized, and readable disclosure statements are urgently needed at the point of influential data presentation.

## Notes

Supplement. **eMethods.** Supplementary Methods
