# Supplementary material for: Assessment of Comprehensibility of Industry Conflicts of Interest and Disclosures by Multiple Sclerosis Researchers at Medical Conferences
Source: JAMA Netw Open. 2021 Apr 2;4(4):e212167. doi: 10.1001/jamanetworkopen.2021.2167 (PMC8019098; doi:10.1001/jamanetworkopen.2021.2167)
Supplement: Supplement. — eMethods. Supplementary Methods [file jamanetwopen-e212167-s001.pdf]

Supplemental Online Content

Martin SJ, Hunt DPJ. Assessment of comprehensibility of industry conflicts of interest and disclosures by multiple sclerosis researchers at medical conferences. *JAMA Netw Open.* 2021;4(4):e212167. doi:10.1001/jamanetworkopen.2021.2167

eMethods. Supplementary Methods

This supplementary material has been provided by the authors to give readers additional information about this work.

© 2021 Martin S-J et al. *JAMA Network Open.*

eMethods. Supplementary Methods

This study involved publicly available data where participants are not identified, and therefore ethical approval was not required.

Both authors independently reviewed platform presentations (publicly and freely available https://onlinelibrary.ectrims-congress.eu/ectrims/, most recently accessed June 9th 2020), and recorded the length of time the disclosure slide was displayed. Data acquisition and analyses were performed March-July 2020. Scientific Sessions (108), Educational sessions (48), Free Communications (19), Hot Topics (29), Plenary Sessions (6), Satellite Symposia (17) and Young Scientific Investigator Sessions (13) were included. The length of time the disclosure slide was displayed was timed by one author using the video player time stamp and by the second author using a stopwatch. Words within the disclosure slide were manually counted. Microsoft Word “wordcount” function was used for longer disclosures (100+ words).

The declared number of conflicts of interest was determined by counting the number of healthcare companies that the presenting individual reported a paid interaction with. Conflicts of interest for the presenting author only were counted. Conflicts of interest for other authors were not counted. If a company was named more than once this was counted as a single conflict. For example if a travel expenses and consultancy fees from a single company were both declared, this was counted as a single conflict. Where a subsidiary company was named alongside the parent company this was considered a single conflict. Where clarification was required, the conflicts of interest were checked against the supplementary information provided by the conference materials and other sources of information, including https://openpaymentsdata.cms.gov. A presentation was only

© 2021 Martin S-J et al. *JAMA Network Open.*

included in the analysis if the conflicts could be unambiguously identified using this approach. A disclosure slide was classified “readable” if the total number of words in the slide could be read in full at average reading speed (3.8 words per second). If there was no dedicated disclosure slide for a platform presentation, this was classified as “not readable”, and the time of the disclosure slide recorded as zero.

Presentations were delivered by individuals from Europe (165), North America (61), South America (4), Middle East (2), Asia/Australia (8). One hundred and four presentations were delivered by women and 136 by men.

The personal industry payments of the US physicians for the years 2016, 2017 and 2018 were reviewed using ‘general payment’ data available from https://openpaymentsdata.cms.gov. This spanned the three year disclosure period covered by the presentation.

Statistical analyses, descriptive statistics and linear regression were performed using Prism version 8. Correlation was calculated using Spearman’s rank correlation coefficient. Comparison of readable/unreadable proportions between CoI groups was performed using a Chi-squared test for trend. This study was prepared using reference to STROBE reporting guidelines for cross-sectional studies http://www.equator-network.org/reporting-guidelines/strobe/

© 2021 Martin S-J et al. *JAMA Network Open.*
